# A cell-free system for production of 2,3-butanediol is robust to growth-toxic compounds

**DOI:** 10.1016/j.mec.2019.e00114

**Published:** 2019-11-20

**Authors:** Jennifer E. Kay, Michael C. Jewett

**Affiliations:** a Department of Chemical and Biological Engineering, Northwestern University, Evanston, IL, 60208, USA; b Chemistry of Life Processes Institute, Northwestern University, Evanston, IL, 60208, USA; c Center for Synthetic Biology, Northwestern University, Evanston, IL, 60208, USA; d Robert H. Lurie Comprehensive Cancer Center and Northwestern University, Chicago, IL, 60611, USA; e Simpson Querrey Institute, Northwestern University, Chicago, IL, 60611, USA

## Abstract

The need for sustainable, low-cost production of bioenergy and commodity chemicals is increasing. Unfortunately, the engineering potential of whole-cell catalysts to address this need can be hampered by cellular toxicity. When such bottlenecks limit the commercial feasibility of whole-cell fermentation, cell-free, or *in vitro*, based approaches may offer an alternative. Here, we assess the impact of three classes of growth toxic compounds on crude extract-based, cell-free chemical conversions. As a model system, we test a metabolic pathway for conversion of glucose to 2,3-butanediol (2,3-BDO) in lysates of *Escherichia coli*. First, we characterized 2,3-BDO production with different classes of antibiotics and found, as expected, that the system is uninhibited by compounds that prevent cell growth by means of cell wall replication and DNA, RNA, and protein synthesis. Second, we considered the impact of polar solvent addition (*e.g.,* methanol, n-butanol)*.* We observed that volumetric productivities (g/L/h) were slowed with increasing hydrophobicity of added alcohols. Finally, we investigated the effects of using pretreated biomass hydrolysate as a feed stock, observing a 25% reduction in 2,3-BDO production as a result of coumaroyl and feruloyl amides. Overall, we find the cell-free system to be robust to working concentrations of antibiotics and other compounds that are toxic to cell growth, but do not denature or inhibit relevant enzymes.

## Highlights

• Assessed impact of growth toxic compounds on cell-free biochemical conversions.
• Varied concentrations of antibiotics, polar solvents, and pretreated biomass hydrolysates.
• Tested conversion of glucose to 2,3-butanediol in lysates of *Escherichia coli*.
• Observed that cell-free systems are robust to compounds that are toxic to cell growth.

## 1 Introduction

Metabolic engineering efforts to use microorganisms as cellular factories have made considerable progress. In recent years, for example, new commercial enterprises such as DuPont’s bio-1,3-propanediol and cellulosic ethanol facilities and Novamont’s 1,4-butanediol plant along with many others (Choi et al., 2015) are beginning to transform the bioeconomy (Clomburg et al., 2017). Unfortunately, engineering microorganisms with sets of enzymes that can convert readily available molecules to desired products with performance characteristics that meet business needs remains difficult (Keasling, 2012; Nielsen et al., 2014). Common problems afflicting the current state-of-the-art include constraints arising from the fact that microbial adaptation, growth, and survival objectives are often opposed to the overproduction and release of a single biomolecular product, build-up of toxic intermediates, and synthesis of toxic products (Alper and Avalos, 2018; Nielsen and Keasling, 2016). Bypassing constraints imposed by having to maintain cell viability, cell-free systems have emerged as an alternative approach (Carlson et al., 2012; Silverman et al., 2019).

Over the last decade, the use of cell-free systems for metabolic engineering, both purified systems and crude extracts, has generated significant interest, in part because of advantages that include: efficient use of substrates, lack of viability constraints, controlled reaction conditions, easy sampling, and tolerance to growth-toxic substances (Dudley et al., 2015; Rollin et al., 2013). Recent innovations in purified systems have improved the cost and reaction lifetime. For example, the development of a “molecular purge valve” and “molecular rheostat” allow regulation of the supply of reducing equivalents and ATP (Korman et al., 2014, 2017; Opgenorth et al., 2014, 2016, 2017). This enables cell-free systems that run for multiple days and produce high titers of isobutanol, monoterpenes (*e.g.,* limonene, pinene and sabinene), and cannabinoid precursors cannabigerolic acid and cannabigerovarinic acid (Valliere et al., 2019). Unfortunately, the need for purification of biosynthetic enzymes can still be cost-prohibitory.

Crude extract-based cell-free biochemical conversions avoid the need for purifying individual enzymes and have the added benefit of native energy and cofactor regeneration (Dudley et al., 2015; Guterl et al., 2012; Karim et al., 2016; Rollin et al., 2013). For example, we showed that glycolysis in crude *Escherichia coli* lysates powered the production of 2,3-butanediol (2,3-BDO) with high yields, high titers (>80 ​g/L), and high volumetric peak productivities (11.3 ​± ​0.1 ​g ​L^−1^·h^−1^) (Kay and Jewett, 2015). While crude extract-based systems have been shown to catabolize a range of carbon substrates to power biochemical synthesis (Karim et al., 2018; Wang and Zhang, 2009), practical substrates like biomass hydrolysate prepared by various methods contain growth-toxic byproducts (Piotrowski et al., 2014) and have not been evaluated for their impact on crude extract-based cell-free biochemical conversions.

**Fig. 1:**
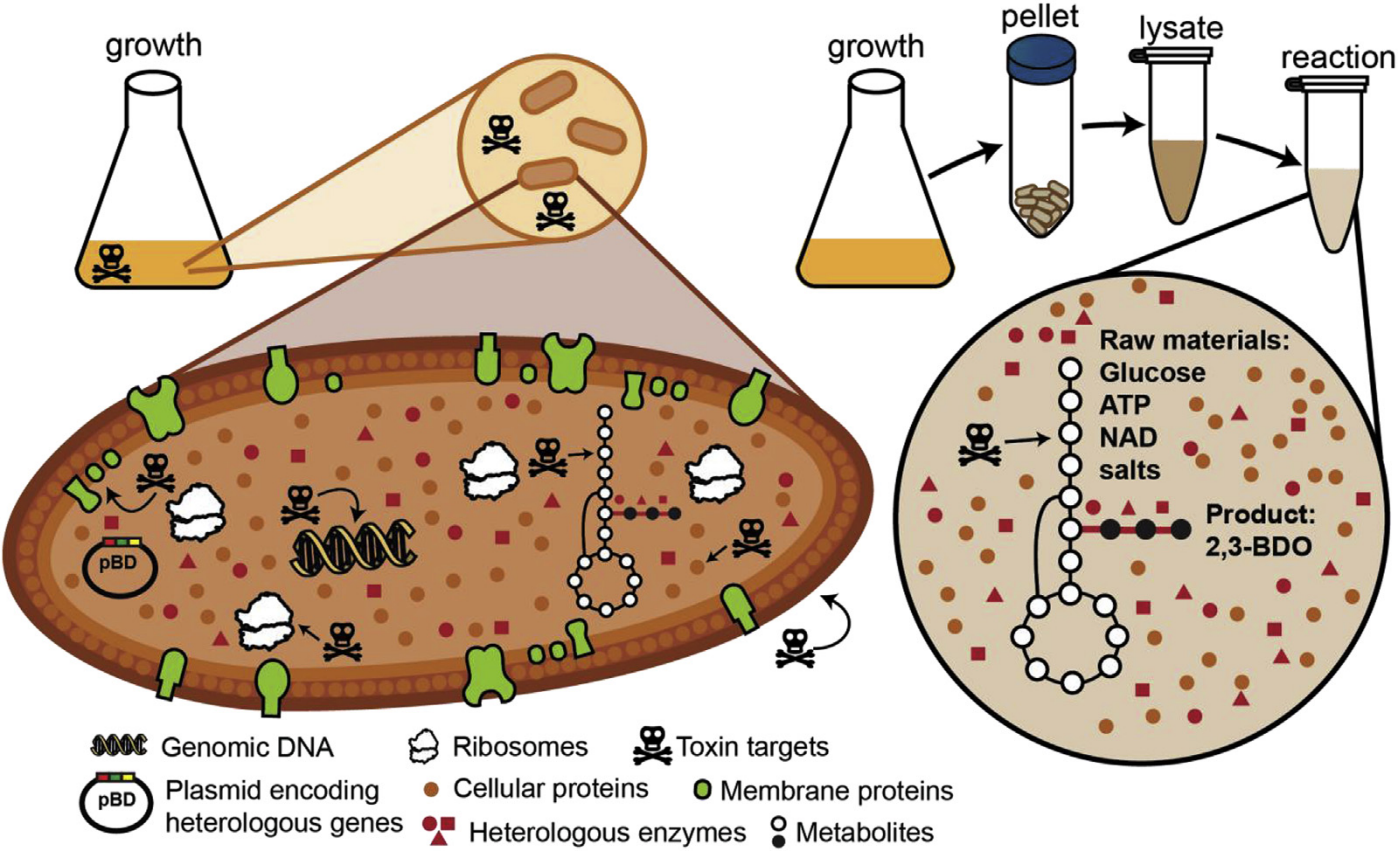
**Crude extract-based cell-free biochemical conversions are more tolerant to growth-toxic compounds.** A traditional fermentation process is represented on the left. Growth-toxic substances may be present in the growth media or may be produced as part of a desired pathway. Live cells have reduced or absent productivity when the cell wall, transporters, DNA synthesis, ribosomes, or enzymes are affected. A cell-free process is illustrated on the right. A standard growth media is used for production of catalyst. The cell-free reaction is only affected by substances that impact required metabolic activity. Note that T7 RNA polymerase and DNA encoding the pathway are omitted from the right panel because they are not part of the *in vitro* reaction to produce 2,3-butanediol (all enzymes are produced in the whole cell prior to lysis; left panel pBD plasmid).

In this work, we set out to evaluate crude extract-based cell-free system tolerance to a variety of growth-toxic substances (Fig. 1), using cell-free 2,3-BDO synthesis as a model. The key idea was to identify conditions that are toxic to live cells but tolerated by the *E. coli* crude lysate system. We specifically assessed the titer and production rate of 2,3-BDO by varying types of toxic compounds (*i.e.* antibiotics, polar solvents, and pretreated biomass hydrolysates) and their concentrations. First, a panel of antibiotics with well characterized mechanisms of growth-toxicity was tested. Second, we examined the effect of several polar solvents, which may be produced as desired products (*e.g.,* butanol, acetone, ethanol), supplemented to increase solubility of some compounds, or added as part of an extraction or other processing method. Finally, we replaced glucose with biomass hydrolysate as the reaction substrate, showing a practical application of cell-free toxicity tolerance. Biomass hydrolysate prepared by various methods contain growth-toxic byproducts (Piotrowski et al., 2014), many of which were also profiled individually. This work provides evidence that cell-free systems are more tolerant to toxic substances than whole cells and sets the stage to consider crude extract-based cell-free transformations when producing such products.

## 2 Materials and methods

### 2.1 Chemicals

BD Bacto™ brand media components tryptone and yeast extract were obtained from BD Biosciences (San Jose, CA). Carbenicillin was obtained from IBI Scientific (Peosta, IA) and isopropyl-β-D-thiogalactoside (IPTG) from Santa Cruz Biotechnology (Dallas, TX). AFEX-pretreated hydrolysate, synthetic hydrolysate, coumaroyl and feruloyl amide samples were kindly provided by Yaoping Zhao and Robert Landick of the Great Lakes Bioenergy Research Center in Madison, Wisconsin. All other reagents are analytical grade from Sigma Aldrich (St. Louis, MO).

### 2.2 Cell-free reactions

*Escherichia coli* BL21(DE3) (NEB) with plasmid pETBCS-alsS-alsD-budC was used to produce the cell extract for this study as previously described (Kay and Jewett, 2015). Reactions were mixed according to Table 1, aliquoted into 25 ​μL in 1.5 ​mL Eppendorf tubes and incubated at 37 ​°C for 2 ​h, which is the middle of the linear range of the reaction from 200 ​mM glucose for this system (Kay and Jewett, 2015). For the 2,3-BDO system, all transcription and translation occur *in vivo* before the cell-free reaction. The key idea is to overexpress the pathway enzymes in cells, which after lysis are then enriched with the pathway. To examine effects on transcription and translation, a second set of reactions were performed with a standard cell-free protein synthesis system (Kwon and Jewett, 2015) with plasmid pJL1-sfGFP (Addgene Plasmid #102634). Each experiment in this study examines the effect of supplementing different types of components to the standard reaction mixture. Polar solvents, especially DMSO, were difficult to deliver in small volumes which resulted in greater variability for those reactions. Reactions are stopped by addition of 0.2 ​M sulfuric acid and precipitated protein is removed by centrifugation at 20,000 x *g* for 15 ​min. Supernatants were stored at −80 ​°C until analysis by HPLC.

**Table 1:** Standard reaction conditions for 2,3-BDO system.

Reaction component	[mM]
Potassium glutamate	130
Magnesium glutamate	12
Ammonium glutamate	10
Potassium phosphate pH 7.2	10
Glucose	200
NAD	1
ATP	1
S30 extract	13 ​mg/mL
Potassium acetate	19.5
Magnesium acetate	4.6
Tris acetate	3.3

### 2.3 Chromatography

High-performance liquid chromatography (HPLC) was used primarily to measure glucose and 2,3-BDO in cell-free reaction supernatant. An Agilent 1260 series HPLC system with refractive index detector (Agilent, Santa Clara, CA) and Aminex HPX 87-H column (Bio-Rad, Hercules, CA) was used with a flow rate of 0.55 ​mL ​min^−1^ 5 ​mM sulfuric acid at 35 ​°C for 25 ​min. A standard calibration was used to determine concentrations.

## 3 Results and discussion

The goal of this work was to assess the impact of growth-toxic compounds on crude extract-based cell-free metabolic conversions. As a model system, we used our previously described approach for the synthesis of 2,3-BDO (Kay and Jewett, 2015). In this system, we engineered a single strain of *E. coli* to express three pathway enzymes necessary to make 2,3-BDO from the pyruvate node of glycolysis, which includes acetolactate synthase (ALS) and acetolactate decarboxylase (ALDC) from *Bacillus subtilis*, and 2,3-BDO dehydrogenase (BDH) from *Klebsiella pneumoniae*. Following overexpression in the extract source strain, cells are lysed to create an extract pre-enriched with the enzymes necessary for 2,3-BDO production. Upon the addition of glucose and catalytic amounts of cofactors NAD+ and ATP, endogenous glycolytic enzymes convert glucose to pyruvate, and then the heterologous pathway completes conversion to 2,3-BDO. We hypothesized that this crude lysate system would be resilient to growth-toxic compounds that have a mechanism of action tied to growth but not biosynthetically-relevant aspects of metabolism. We tested this hypothesis in three ways. First, we assessed the effect of different antibiotics. Second, we explored the impact of different polar solvents. Third, we studied pathway performance when the glucose substrate was replaced with biomass hydrolysates, which are known to inhibit cell growth.

### 3.1 The impact of antibiotics on crude extract-based 2,3-BDO production

**Fig. 2:**
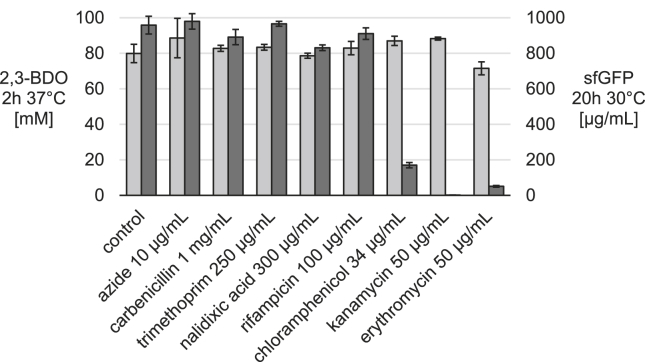
**2,3-BDO synthesis is not affected by antibiotics affecting the electron transport chain, cell wall, DNA synthesis, and ribosome.** 2,3-BDO produced by enriched lysate reactions with glucose after 2 ​h at 37 ​°C (left; light grey). sfGFP produced by CFPS system after 20 ​ ​h ​at 30 ​°C (right; dark grey). 2,3-BDO components according to Table 1 and CFPS as previously described, except with antibiotic additions. Reactions were run in triplicate with error bars representing one standard deviation for n=3. [2,3-BDO] is statistically equivalent to control reactions in the presence of working concentrations of antibiotics that target several systems necessary for cell growth. The CFPS system tolerates all of the panel additions except those that target the ribosome, which is necessary for protein production.

We first chose to screen a panel of antibiotics that have known mechanisms of inhibiting growth in live cells and are effective at low concentrations. We performed cell-free 2,3-BDO production reactions for 2 ​h and cell-free protein synthesis reactions of superfolder green fluorescent protein (sfGFP) for 20 ​h with working concentrations of azide, carbenicillin, trimethoprim, nalidixic acid, rifampicin, chloramphenicol, kanamycin, and erythromycin (Fig. 2). These reactions consisted of lysates pre-enriched with enzymes from the 2,3-BDO pathway, along with substrates, cofactors, and salts designed to mimic the cytoplasmic environment. 2,3-BDO synthesis was not affected by the panel of antibiotics while protein synthesis was only inhibited by compounds targeting the ribosome, which is necessary for translation.

Carbenicillin is an excellent example of a compound which only affects cell growth because it interferes with integration of monomer units into the cell wall rather than destabilization of the membrane via hydrophobic interactions (Kohanski et al., 2010). Carbenicillin did not inhibit either protein synthesis or 2,3-BDO production at up to 10x normal working concentration for *E. coli*. Because the 2,3-BDO system does not require DNA and template DNA is added to the CFPS system either as purified plasmid or amplified linear DNA, as expected the system was not affected by nalidixic acid which targets DNA synthesis. Rifampicins are inhibitors of bacterial RNA polymerases (Kohanski et al., 2010). Neither system is affected by rifampicin, because the 2,3-BDO system only requires metabolic activity and the CFPS system relies on supplemented purified viral T7 polymerase rather than the native RNA polymerase. Our observation that neither system is affected by azide or trimethoprim implies their targets are not key for overall metabolic activity in the lysate. While the electron transport chain has been shown to be present in the lysate via inverted membrane vesicles (Jewett et al., 2008), it is unnecessary in the PEP-dependent CFPS system because ATP regeneration doesn’t rely on oxidative phosphorylation from inverted membrane vesicles (Kim and Swartz, 2001). There should be an excess of ATP produced by glycolysis in the 2,3-BDO system so in this case the function of the electron transport chain is also not necessary. Practical interest in use of antibiotics in cell-free systems is likely limited to lab-scale applications such as selectively de-activating cell-free protein synthesis while allowing enzymatic reactions to proceed and ensuring prevention of microbial growth in long experiments. However, our data do show tolerance to growth toxic compounds.

### 3.2 The impact of polar solvents on crude extract-based 2,3-BDO production

Sometimes desired substrates, intermediates, and products in metabolic pathways for chemical conversions have undesirable bulk effects on enzymes and membranes. For instance, biological systems have been considered for production of polar solvents ethanol, isopropanol, and butanol, yet are often only tolerant to low levels of these compounds. Enzymatic systems are often more tolerant of polar and organic solvents than live cells (Drauz et al., 2012). For example, individual enzymes are often found to have DMSO tolerance of up to 10 or 30% (Drauz et al., 2012). However, the crude lysate system engineered for 2,3-BDO production requires more than ten enzymes to be active, not to mention the need for multiple enzymes that may be involved in cofactor and energy regeneration to be active. We were curious how the system would tolerate polar solvents, given that deactivation of any one necessary enzyme would slow or stop synthesis of a desired product.

**Fig. 3:**
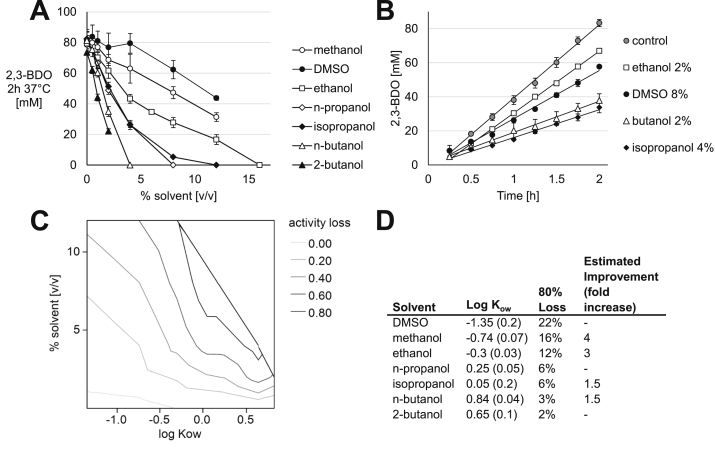
**The rate of 2,3-BDO production is deleteriously impacted by DMSO and alcohols, but tolerates several polar solvents better than growth in standard *E. coli* strains. (A)** 2,3-BDO produced by reactions with addition of varying volumes of solvents after 2 ​h at 37 ​°C. **(B)** 2,3-BDO concentration over the 2-h period for selected points from panel A. The rate is decreased but the reaction proceeds linearly over the course of 2 ​h. Values represent averages (n ​≥ ​3) and error bars represent 1 standard deviation. ​**(C)** Data from panel A represented as equal lines of % of control activity lost as a function of volume added and log K_ow_ (Sangster, 1989). **(D)** Table summarizing log K_ow_, volume limit by linear fit calculation of 80% loss of control activity, and approximate fold-improvement in tolerance over live *E. coli*.

To test the impact of polar solvents spanning a range of hydrophobicity on cell-free 2,3-BDO production, DMSO, methanol, ethanol, n-propanol, isopropanol, n-butanol, or 2-butanol were each added individually at up to 16 percent of reaction volume (Fig. 3A). We then continued to collect full time course data for a reduced set of conditions (Fig. 3B). Specifically, we probed whether the reductions in titer we observed were due to inactivation over time (decreasing rate) or inhibition of a key step (constant rate). It seems that any deactivation occurs in a general way, with the reactions proceeding linearly over 2 ​h at a lower rate than the control without solvent. It is not clear whether this is a direct effect of solvent on the enzymes, such as by dehydration (Doukyu and Ogino, 2010), or whether a small amount of a key reaction component is being precipitated out of solution with increasing hydrophobicity and volume of solvents.

Hydrophobicity of organic compounds is frequently represented by their partition coefficient between phases of octanol and water (K_ow_). The solvents were tolerated roughly in order of reported log K_ow_, with the greatest additions tolerated for DMSO and methanol and the least for more hydrophobic n- and 2-butanol. While it is not possible to directly compare traditional fermentations to cell-free reactions, the 2,3-BDO reaction proceeds, if slowed, outside of the range tolerated by the live strain used to produce crude lysate. For example, *E. coli* and *Pichia pastoris* are able to grow in the presence of 4% (v/v) but not 10% (v/v) methanol (Ganske and Bornscheuer, 2006; Muller et al., 2015). In the *in vitro* reaction, *E. coli* metabolism is slowed but proceeds well above 10% (v/v) methanol. Typical strains of *E. coli* do not grow well in media containing above 4% (v/v) ethanol, but production of up to 6 ​g/L has been observed in engineered strains (Haft et al., 2014; Luhe et al., 2012; Yomano et al., 1998). While membrane effects are important, recent work has shown effects on transcription and translation to be key to the toxic effect of ethanol on cell growth (Haft et al., 2014). In our study, the rate of 2,3-BDO production in 12% (v/v) ethanol is only one quarter of the rate in normal assay medium, but this is still about twice the solvent concentration that seems to be the current limit for growth of live *E. coli*. In the best isopropanol-producing system to date, an engineered strain of *E. coli* tolerated and was able to produce up to 40 ​g/L isopropanol before a continuous product-removal strategy was necessary for further increase (Inokuma et al., 2010). A step-change to ~35 ​g/L isopropanol early in the culture resulted in stalling of growth, demonstrating a growth-toxicity effect of the product isopropanol versus only product-inhibition of the desired pathway (Inokuma et al., 2010). The *in vitro* 2,3-BDO reaction is slowed but clearly proceeding with 4% (v/v) isopropanol in the assay media. Typical strains of *E. coli* and yeast do not tolerate above 2% (v/v) n-butanol while an engineered strain of *E. coli* and some natural strains of *Lactobacillus* were able to produce or tolerate up to 30 ​g/L and 3% (v/v) (Knoshaug and Zhang, 2009; Shen et al., 2011). In this case the limiting amount of butanol for the engineered fermentation and this cell-free system are similar. Overall, the less hydrophobic compounds were tolerated at a greater fraction of total volume and had larger gains in tolerance over live *E. coli*. Clearly, there are some examples for which removing cell viability constraints allows biosynthetic pathway operation in the presence of increased concentration of solvents.

### 3.3 The impact of biomass hydrolysates on crude extract-based 2,3-BDO production

Many pathways of interest do not involve growth-inhibitory antibiotics or solvents, but they may have toxicity resulting from desired feedstocks. While using pure glucose as a substrate may be suitable for synthesizing high value compounds, molecules produced at large scale must use inexpensive, environmentally, and socially responsible feedstocks. A good example is biomass hydrolysate. Depending on the method used for production, small molecule byproducts like ‘lignotoxins’ present in the hydrolysates can inhibit cell growth by disrupting cellular membranes, inhibiting metabolic enzymes, and draining ATP via efflux pumps (Mills et al., 2009; Piotrowski et al., 2014). While toxic to cells, the ability to use biomass hydrolysate feedstocks as the energy and carbon source for cell-free metabolic reactions would provide a distinct advantage in using cell-free systems for industrial biotechnology. To explore this possibility, we carried out a series of experiments with biomass hydrolysate samples pre-treated by the ammonia fiber expansion process (AFEX) as well as a synthetic recipe with and without identified lignotoxins (Keating et al., 2014) in place of glucose. The goal was to understand how the crude extract-based 2,3-BDO system would tolerate the bulk hydrolysate and specifically the group of suspected toxins.

**Fig. 4:**
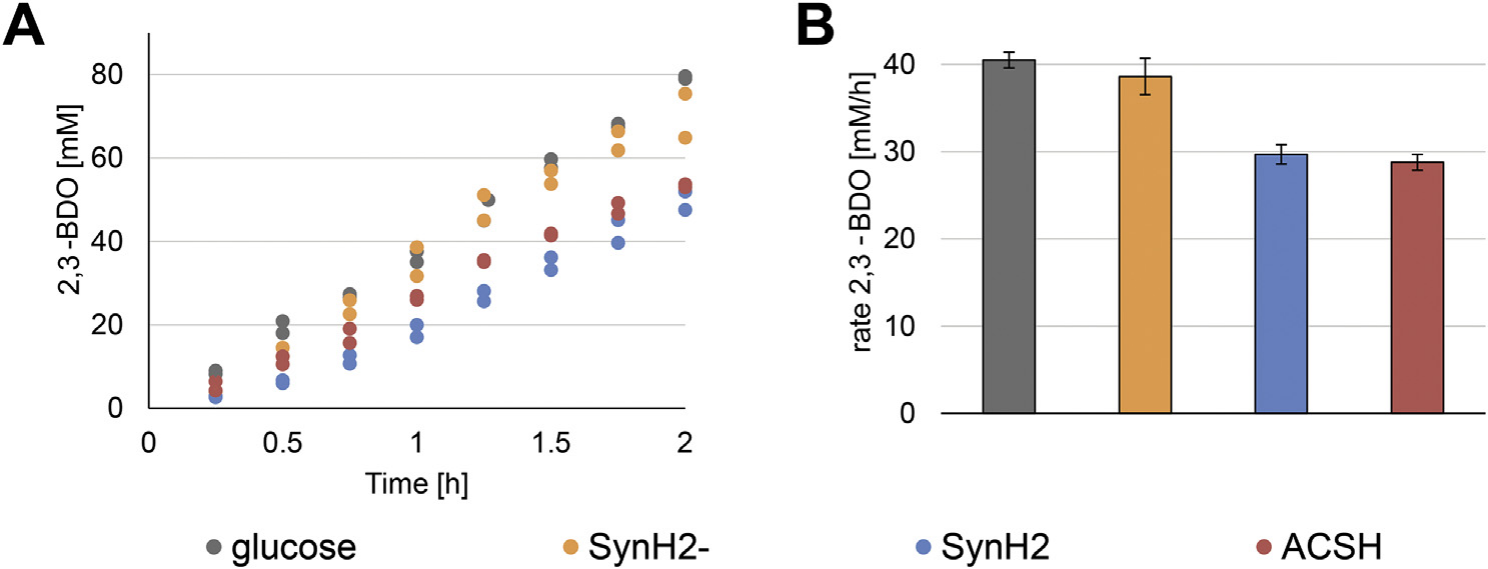
**The conversion of glucose to 2,3-BDO is inhibited by the ‘lignotoxic’ component of the AFEX-pretreated biomass hydrolysate. (A)** Time course of crude extract-based cell-free synthesis of 2,3-BDO. **(B)** Rates and errors indicate mean and standard deviation (S.D.) from n=3 reactions. The hydrolysate is added at 56% by volume which sets the initial glucose concentration equal to the control. Substrates include glucose, AFEX treated hydrolysate (ACSH), synthetic biomass hydrolysate with (SynH2) and without (SynH2-) toxins. Without this group of known toxins, the synthetic hydrolysate recipe is statistically similar to glucose.

To replace glucose with hydrolysate, samples of hydrolysate were first brought to pH 7.0 by addition of potassium hydroxide. Then, a 2-h time course cell-free reaction was performed with these samples and the rate of 2,3-BDO was calculated by linear regression (Fig. 4). The synthetic recipe to match AFEX-pretreated hydrolysate without suspected toxins performs statistically similar to the control. In the synthetic recipe where suspected toxins *p*-coumaric and ferulic acid, 5-hydroxymethyl-2-furaldehyde (HMF) and coumaroyl and feruloyl amides are added, the rate of 2,3-BDO production is decreased by approximately 25%. The genuine hydrolysate performs statistically similar to the synthetic recipe with toxins. Clearly, the reaction of glucose to 2,3-BDO is also inhibited by these lignocellulose-derived toxins. However, we wanted to know which compounds in particular were inhibiting the cell-free metabolic reactions.

**Fig. 5:**
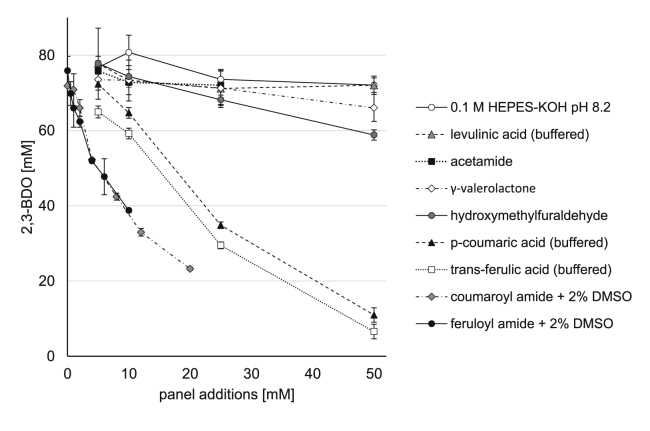
**The coumaroyl and feruloyl amides are responsible for the majority of the decrease in 2,3-BDO production at the relevant concentrations contained in the hydrolysate.** Data are the 2,3-BDO produced after 2 ​h at 37 ​°C with addition of panel components up to 50 ​mM or equal volume addition of 0.1 ​M HEPES-KOH buffer at pH 8.2. The amides were added from stock solutions in DMSO, but extra DMSO was added to keep the concentration at 2% by volume for the entire panel.

To determine which growth-toxic compounds from the biomass hydrolysates were inhibiting the cell-free system, we screened a panel of candidate compounds from the synthetic hydrolysates individually at a wide range of concentrations (Fig. 5). Specifically, each compound was added to cell-free reactions utilizing glucose as a primary feed source. Growth-toxic compounds found in hydrolysates produced by different methods were included to determine if another pretreatment option might be more suitable. The reaction was inhibited by *p-*coumaric acid and trans-ferulic acid, but the effect should be negligible at the concentration of these compounds contained in AFEX pretreated hydrolysate, 2.10 and 0.71 ​mM respectively (Keating et al., 2014). The 2,3-BDO reaction was uninhibited by levulinate up to 50 ​mM. The high tolerance of the reaction to γ-valerolactone (GVL) and hydroxymethylfuraldehyde (HMF) suggest other methods of processing biomass such as dilute acid pretreatment may be preferred for the cell-free system. HMF is typically present at up to 5 ​g/L or 40 ​mM in biomass hydrolysates (Mills et al., 2009), and the IC_50_ concentration for yeast is only about 10 ​mM (Bottoms et al., 2018; Piotrowski et al., 2015). Coumaroyl and feruloyl amides are contained at ~5.5 ​mM each in the synthetic hydrolysate recipe for a final concentration of 3.3 ​mM in the cell-free reaction. At the concentrations of lignotoxins contained in the hydrolysate samples, coumaroyl and feruloyl amide appear to be primarily responsible for the observed decrease in activity.

The reason the reaction is affected by coumaroyl and feruloyl amides is not readily apparent. In live cells, these are thought to inhibit growth by increasing expression of efflux pumps (wasting cellular energy by toxin export) and interfering with purine and pyrimidine synthesis (Keating et al., 2014; Pisithkul et al., 2015). ^13^C-labeling experiments showed rapid inhibition of multiple metabolic enzymes, effects we would also expect to see in our system if the activities were relevant to glycolysis and 2,3-BDO pathway activity. However, direct inhibition of metabolic enzymes appears primarily to effect purine and pyrimidine synthesis, which should not be involved in the conversion of glucose to 2,3-BDO. Though it was not thought to be the most important problem for growth in cells, both papers note a change in the pyruvate metabolic node. Keating et al. found buildup of pyruvate in their experiments in the presence of the toxic group of compounds. In C^13^-labeling experiments, Pisithkul et al. (2015) found a decrease in the synthesis of valine, which comes off of the pyruvate node through acetolactate synthase, the first step between pyruvate and 2,3-BDO. Keating et al. (2014) also found a decrease in the concentration of glycolytic intermediate fructose-6-phosphate. Since we did not see accumulation of pyruvate, we postulate that the cell-free reaction is slowed by inhibition in the glycolytic pathway or metabolic activity related to cofactor balance. Of note, many of the observed effects of inhibitors appear to be principally mediated by transcriptional regulation (Keating et al., 2014; Pisithkul et al., 2015), which would not be observed in our system since the 2,3-BDO pathway is already expressed before substrate and inhibitor addition.

## 4 Summary

In this work, we demonstrate that cell-free production of 2,3-BDO is robust to growth toxic compounds. Specifically, we found that the crude extract-based cell-free system is robust to growth-toxic compounds that act on the cell wall, ribosome, and DNA and RNA synthesis as evident by our antibiotic panel test. In addition, solvents affect enzyme stability and decrease the biosynthetic reaction rate, though the reaction appears to tolerate much greater concentrations of some substances than is possible in cells, such as DMSO and methanol. This could be beneficial for working with less soluble substrates, intermediates, or products. Finally, we observed that many growth-toxic compounds present in biomass hydrolysates, an economical and socially and environmentally responsible source of sugars, are better tolerated in the cell-free environment than by live cells. Beyond the variables studied here, future works could explore additional environmental factors that can adversely affect growth, such as broader pH and temperature ranges. Taken together, our data join an emerging set of demonstrations that highlight the potential to use a cell-free approach for biochemical conversions when cell viability constraints may be limiting. Looking forward, we anticipate that cell-free systems will enable use of pathways and reaction compositions that are otherwise difficult to achieve in live strains to advance industrial biotechnology.

## Author contributions

JEK designed the project, performed all the experiments, and analyzed the data. MCJ provided a supervisory role. Both authors wrote the manuscript.

## Declaration of competing interest

None.
